# Identification and validation of a prognostic model based on four genes related to satellite nodules in hepatocellular carcinoma

**DOI:** 10.1038/s41598-024-66610-z

**Published:** 2024-07-07

**Authors:** Feng Liu, Tinghua Yan, Dan Cui, Jinhua Jiang

**Affiliations:** 1 Department of Interventional Oncology, Renji Hospital, School of Medicine, Shanghai Jiaotong University, Shanghai, China; 2 The First Clinical Medical College of Jinan University, Guangzhou, China

**Keywords:** Hepatocellular carcinoma, Satellite nodules, Prognostic model, Satellite nodules-related genes, Tumor-infiltrating lymphocytes, Cancer, Cancer prevention

## Abstract

Satellite nodules is a key clinical characteristic which has prognostic value of hepatocellular carcinoma (HCC). Currently, there is no gene-level predictive model for Satellite nodules in liver cancer. For the 377 HCC cases collected from the dataset of Cancer Genome Atlas (TCGA), their original pathological data were analyzed to extract information regarding satellite nodules status as well as other relevant pathological data. Then, this study employed statistical modeling for prognostic model establishment in TCGA, and validation in International Cancer Genome Consortium (ICGC) cohorts and GSE76427. Through rigorous statistical analyses, 253 differential satellite nodules-related genes (SNRGs) were identified, and four key genes related to satellite nodules and prognosis were selected to construct a prognostic model. The high-risk group predicted by our model exhibited an unfavorable overall survival (OS) outlook and demonstrated an association with adverse worse clinical characteristics such as larger tumor size, higher alpha-fetoprotein, microvascular invasion and advanced stage. Moreover, the validation of the model's prognostic value in the ICGC and GSE76427 cohorts mirrored that of the TCGA cohort. Besides, the high-risk group also showed higher levels of resting Dendritic cells, M0 macrophages infiltration, alongside decreased levels of CD8^+^ T cells and γδT cells infiltration. The prognostic model based on SNRGs can reliability predict the OS of HCC and is likely to have predictive value of immunotherapy for HCC.

## Introduction

Hepatocellular carcinoma (HCC) is one of the leading cancers worldwide and is the only major cancer for which death rates have not improved over the last 10 years^1,2^. The pathological and molecular heterogeneity of HCC can lead to ineffective treatment outcomes and present formidable obstacles in the pursuit of administering precision medicine^3–5^. In terms of pathological heterogeneity, patients exhibiting satellite nodules, larger tumor sizes, microvascular invasions, limited differentiation, and advanced stages generally exemplify rapid tumor growth patterns and a poor prognosis^6–9^. For molecular heterogeneity, a number of studies have found that genetic heterogeneity reflects the degree of malignancy of the tumor and the sensitivity to treatment^7,10^. Given that pathologic and molecular heterogeneity collectively influence the prognosis of HCC, there arises a need to establish a model capable of discerning HCC with dual-dimensional heterogeneity.

Satellite nodules refer to small tumor lesions located in proximity to the primary tumor, which are considered to arise from intrahepatic metastasis, and the presence of satellite nodules is one of the indicators for tumor invasiveness^11^. Numerous studies have shown that satellite nodules increasing the incidence of postoperative recurrence^12–14^. And recently, detection of satellite nodules based on magnetic resonance imaging (MRI) has shown robust performance in stratifying patients with early and overall tumor recurrence^15^. Currently, the detection of satellite nodules predominantly relies on pathology and imaging, and there are still no recommended satellite nodules-related genes molecular markers to predict the prognosis of HCC. This limitation hampers the ability to identify tumors with a propensity for developing satellite nodules, thereby constraining the utility of satellite nodules in guiding the diagnosis and treatment strategies for liver cancer.

In this study, we used TCGA to explore novel satellite nodules-related genes, analyses the associations between these genes and clinical characteristics and established a risk scoring model for predicting the prognosis of HCC, with the aim of providing suitable treatments for patients with HCC.

## Materials and methods

### Data acquisition and preprocessing

The RNA expression data, pathological data and clinical data of The Cancer Genome Atlas liver hepatocellular carcinoma cohort (TCGA LIHC) were downloaded from the official website of Genomic Data Commons using R package “TCGAbiolinks”. Satellite nodules and tumor size were extracted from original pathological data in PDF format. The information was retrieved by two individuals separately, then verified by one person. When data conflicts occurred, the original file should be further verified. A total of 50 cancer tissues with satellite nodules, 113cancer tissues without satellite nodule, and 58 normal tissues were collected. Liver Cancer—RIKEN, JP Project from International Cancer Genome Consortium (ICGC LIRI-JP) transcriptomic expression data and GSE76427.were downloaded as a validation cohort. For normalization, gene expression quantified with fragment per kilobase million (FPKM) was transformed into transcripts per million (TPM) values and processed by log2(value + 1) for all samples before further analysis. The overall analysis workflow is presented in Supplementary Fig. 1

### Identification of differential expressed satellite nodules-related genes in TCGA cohort

Cancer tissues with or without satellite nodules were used for the difference analysis with normal tissues to obtain relatively differentially expressed genes, respectively. Cancer tissues with satellite nodules compared with cancer tissue without satellite nodules were used for analysis to obtain absolute differential expression genes. The Wilcox test was used to identify differentially expressed genes (DEGs) according to the criteria of |log 2 (fold change) |> 1 and false discovery rate (FDR) < 0.05.

### Go and KEGG analysis

To identify the genes that promote satellite nodules development, 253 up-regulated DEGs were selected to further analysis. Gene ontology (GO) enrichment analyses was performed by R package “clusterProfiler”, “org.Hs.eg.db” and “enrichplot”. Kyoto Encyclopedia of Genes and Genomes (KEGG) pathway enrichment analyses by http://kobas.cbi.pku.edu.cn/.

### Prognostic signature development and evaluation for HCC

Univariate Cox regression analysis identified hundreds of genes significantly related to the OS of patients with HCC, with a cutoff value of *p* < 0.05. By matching DEGs obtained from comparison between HCC tumor tissues with or without satellite nodules, 8 SNRGs were selected to perform the Least Absolute Shrinkage and Selection Operator (LASSO) analysis, using R package “glmnet” and “foreign”. After LASSO analysis was utilized to reduce the genes of the model and limit the complexity of solving the problem of overfitting, Cox regression analysis war perform to construct the predictive model. The risk score of each patient with HCC was calculated using the following formula: risk score = expression level of gene a * coefficient a + expression level of gene b * coefficient b + expression level of gene c * coefficient c + …… + expression level of gene n * coefficient n. R package “survival”, “survminer”, “ggrisk”, “pROC”, “timeROC” and “ggDCA” were use to reflect the sensitivity and specificity of the signature. After incorporated clinical parameters into the model, R package “rms” was used to create a Create nomogram.

### Clinical characteristics of the signature

To evaluate each SNRGs in model, http://gepia2.cancer-pku.cn and R package “ggpubr” were used to distinguish survival time and clinical parameters grouped by SNRGs expression level, respectively. Tissue type, with/without satellite nodules, tumor size, MVI, AFP level, ECOG score, Child–Pugh score, tumor stage, tumor grade and stage were included. We applied the Wilcoxon test for evaluation. Besides, tumor stage, tumor grade and stage grouped by risk score also evaluated by Wilcoxon test.

### External validation of the genes in the prognostic gene signature

The ICGC LIRI-JP and GSE76427 datasets were used to validate gene signatures. Each patient's risk score was calculated using the above method. A Kaplan–Meier curve was constructed to test the predictive value of the gene signature. The ROC curve was made by above method.

### The correlation between immune infiltration and SNRGs:

The correlation between immune infiltration and each SNRG was performed by https://cistrome.shinyapps.io/. The correlation between immune infiltration and risk score was performed by R package “immunedeconv” and “CIBERSORT”.

### Statistical analysis

In this study, R software v4.2.0 and GraphPad Prism 8 were used for the statistical analysis of the experimental data. The relationship between satellite lesions and clinical characteristics was examined using the chi-square test. For comparison between the two samples, data with normal distribution and uniform variance were analyzed using Student's t-test; data with uneven variances were analyzed using the Wilcox test. For comparison between the three samples, data with normal distribution and uniform variance were analyzed using variance test; data with uneven variances were analyzed using the Kruskal–Wallis-test. Statistical significance was set at *p* < 0.05.

### Ethics approval and consent to participate

The authors take responsibility for all aspects of the research, ensuring that any inquiries regarding the accuracy or integrity of any part of the study are thoroughly examined and resolved. This article does not contain any studies with human or animal subjects.

### Consent for publication

All authors have unanimously agreed to the publication of this paper.

## Results

### Clinical characteristics of HCC patients

The crucial clinical information which had significant impact on survival was sourced from the TCGA official website, including grade, stage, T-stage, ECOG score, AFP and MVI. Besides, satellite nodules and tumor size were extracted from original pathological data stored in TCGA website in PDF format. Among 377 patients with HCC, a total of 50 cancer tissue samples with unmistakable satellite nodules, 113 cancer tissue samples clearly without satellite nodules. The assessment of tumor size revealed a median of 6 (3.5–10) cm, with a range spanning from 1.2 to 25 cm. The detail of each clinical parameter was shown in Supplementary File 1. Further analysis revealed a close correlation between satellite lesions and key pathological factors in clinical settings, including tumor size, stage, T-stage, and MVI (Table 1).

**Table 1:** The clinical characteristics of patients with SN or without SN.

Clinical characteristic			
	With SN (n = 50)	Without SN (n = 113)	*p*
Tumor size			
< 3 cm	1	28	< 0.0001
3–7 cm	17	62	
> 7 cm	32	22	
Unknown	0	1	
Grade			
G1–G2	29	75	0.3051
G3–G4	21	38	
Unknown	0	0	
Stage			
I–II	20	106	< 0.0001
III– IV	27	5	
Unknown	3	2	
T-stage			
T1–T2	21	106	< 0.0001
T3–T4	29	6	
Unknown	0	0	
ECOG socre			
0–1	35	90	0.7579
2–4	3	11	
Unknown	12	12	
AFP			
< 20 ng/ml	18	60	0.5056
20–400 ng/ml	8	17	
> 400 ng/ml	10	21	
Unknown	14	15	
MVI			
Micro	17	28	0.0054
Macro	4	1	
None	22	78	
Unknown	4	0	

### Genes with differential expression between satellite nodules-positive and satellite nodules-negative HCC tissues

**Figure 1:**
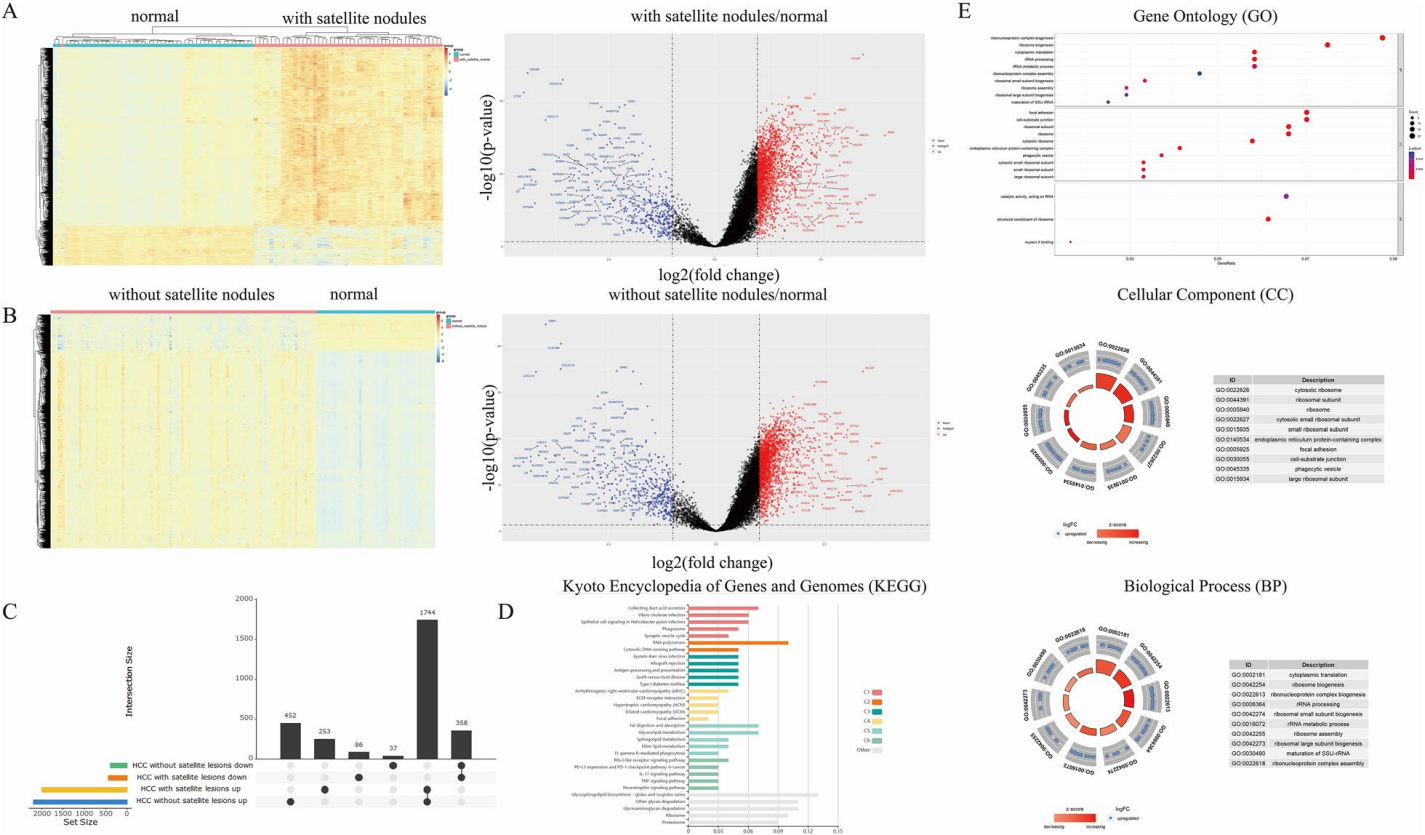
The DEGs and functional analysis. (**A**) The heatmap and the volcano plot of DEGs between HCC tissues with satellite nodules and paracancerous normal tissues. (**B**) The heatmap and the volcano plot of DEGs between HCC tissues without satellite nodules and paracancerous normal tissues. (**C**) The upset diagram of DEGs between HCC with satellite nodules and HCC without satellite nodules. (**D**) Kyoto Encyclopedia of Genes and Genomes (KEGG) analysis. The length of each column represents the count of genes. (**E**) Gene Ontology (GO) enrichment analysis. The size of each circle represents the count of genes; the shade of color represents the *p* value.

To acquire the gene expression profile distinctions between tumors with satellite nodules and those without, we conducted separate comparisons of satellite nodules-positive cancer tissues and satellite nodules-negative tissues with normal tissues. The results of the difference analysis showed that HCC samples can be clearly distinguished from normal tissues (Fig. 1A left, Fig. 1B left). The volcano plot illustrated a distribution of 445 down-regulated DEGs and 1998 up-regulated DEGs in satellite nodules-positive cancer tissues, alongside 396 down-regulated DEGs and 2197 up-regulated DEGs in satellite nodules-negative cancer tissues (Fig. 1A right, Fig. 1B right). To further distinguish the differential expression between two types of HCC, upset diagram was mapped between two DEGs signatures. Specifically, in HCC with satellite lesions, 253 DEGs were identified as up-regulated (Supplementary File 2), while 86 DEGs were down-regulated exclusively (Fig. 1C). To discern genes potentially contributing to the development of satellite nodules, the subset of 253 up-regulated DEGs was selected for further analysis.

### Functional analysis of up-regulated satellite nodules-related DEGs

GO enrichnment analysis and KEGG analysis of 253 up-regulated DEGs were performed using R package and online tool. In cellular component processes (CC), the SNRGs were mainly involved in focal adhesion and cell-substrate junction (Fig. 1E), which involved in epithelial-mesenchymal transition (EMT) and precancerous cell invasion^16,17^. Regarding the pathways associated with Satellite Nodules-Related Genes (SNRGs), the predominant pathways encompassed cell adhesion and extracellular matrix-related pathways, metabolism-related pathways, and pathways relevant to tumor immunity (Fig. 1D).

### Construction and identification of prognostic models

**Figure 2:**
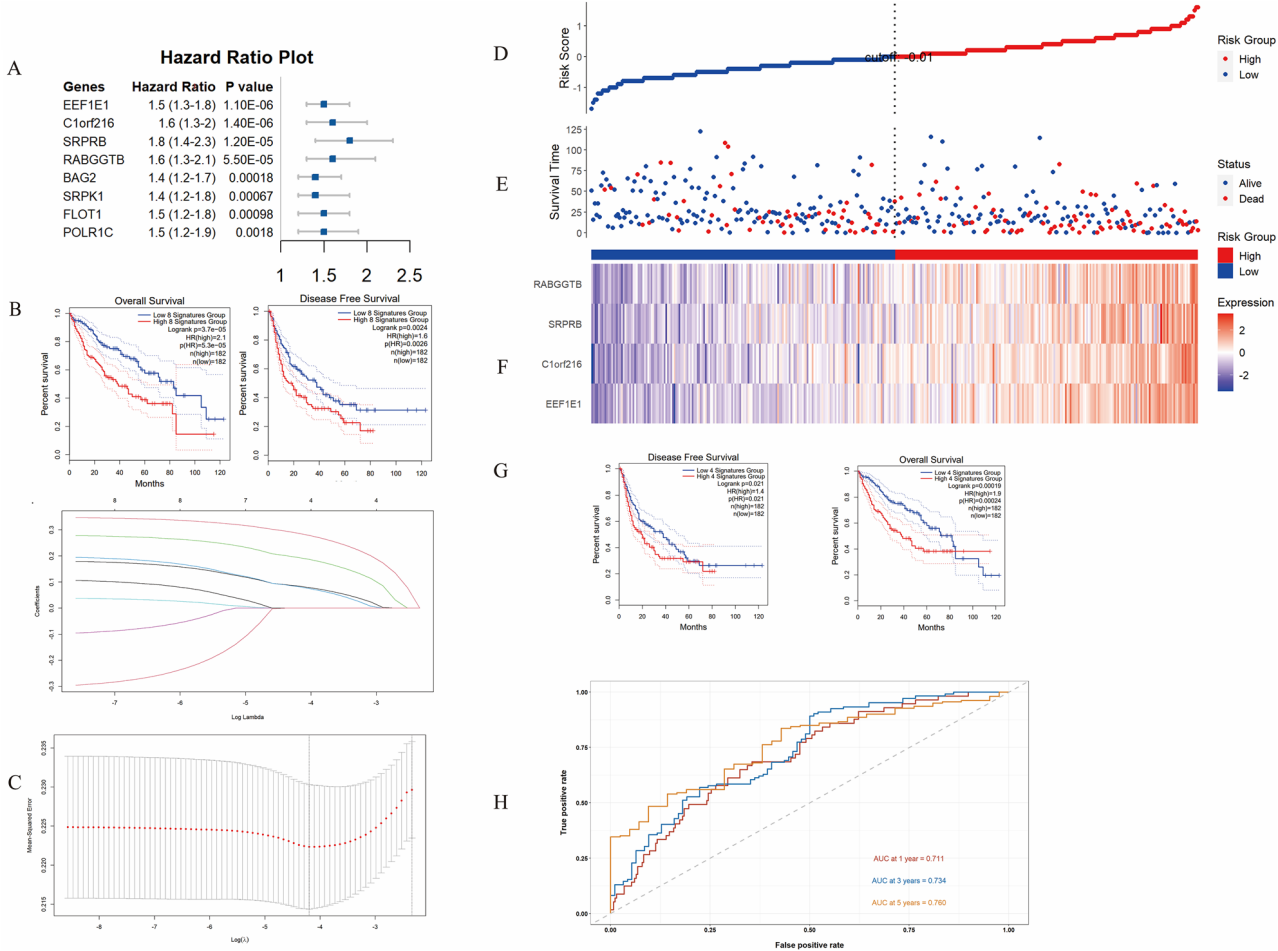
Construction and evaluation prognostic model based on SNRGs in TCGA cohort. (**A**) 8 SNRGs significantly associated with OS and satellite nodules of patients with HCC. (**B**) Kaplan–Meier plot for OS and DFS in high- or low-risk group stratified by 8 SNRGs (*p* = 0.000037 and* p* = 0.0024 respectively). (**C**) The result of LASSO regression for 8 SNRGs. (**D**) The risk score of each patient with HCC. (**E**) The patient survival based on the risk score. (**F**) The heat map of the three SNRGs in the high-risk group and the low-risk group. (**G**) Kaplan–Meier plot for DFS and OS in high or low risk group based on prognostic model (*p* = 0.021 and* p* = 0.00024 respectively). (**F**) Receiver operating characteristic (ROC) curve analysis for the prognostic value of the prognostic model for 1, 3and 5 years survival. Data from TCGA (median risk score as the cut-off value). AUC: area under the curve.

Univariate Cox regression analysis was used to determine the SNRGs correlated with patient survival, in conjunction with performing an absolute comparison of gene expression between tumor tissues with satellite nodules (SN+) and tumor tissues without satellite nodules (SN-) (Supplementary File 3). A total of 8 SNRGs were detected that exhibited a potential significant association with patient survival (Fig. 2A). The clinical outcomes, specifically OS could be clearly distinguished by the expression model consisted by these eight SNRGs (*p* = 0.000037, HR = 2.1), as well as DFS (*p* = 0.0024, HR = 1.8) (Fig. 2B). LASSO regression was performed to mitigate the risk of overfitting among the eight genes. Subsequently, Cox regression analysis was conducted to formulate a prognostic model centered on the presence of satellite nodules. (Fig. 2C, Table 2). Among eight SNRGs, four key genes (RABGGTB, SRPRB, C1orf216 and EEF1E1) were selected to construct the prognostic model (Fig. 2B, Table 2). The risk score formula for the model was established as follows: risk score = (0.2455396 × EEF1E1) + (0.2837982 × C1orf216) + (0.1845994 × SRPRB) + (0.164889 × RABGGTB).

**Table 2:** Four key SNRGs (EEF1E1, C1orf216, SRPRB and RABGGTB) were selected after Cox regression analysis.

SNRGs	Coefficient
EEF1E1	0.2455396
C1orf216	0.2837982
SRPRB	0.1845994
RABGGTB	0.164889

The results showed the risk score distribution in TCGA cohort (Fig. 2D), patient survival time distribution based on risk score (Fig. 2E) and the heat map of four SNRGs of each patient (Fig. 2F). Additionally, the Log-Rank test substantiated the efficacy of this prognostic model in effectively stratifying DFS (*p* = 0.021, HR = 1.4) and OS (*p* = 0.00019, HR = 1.9) for individuals with HCC across the high and low-risk score groups (Fig. 2G). Furthermore, the ROC curve showed that the AUC values were 0.711 (1 year), 0.734 (3 years) and 0.760 (5 years), which indicated that the model performed well in predicting the survival rate of patients with HCC (Fig. 2H).

**Figure 3:**
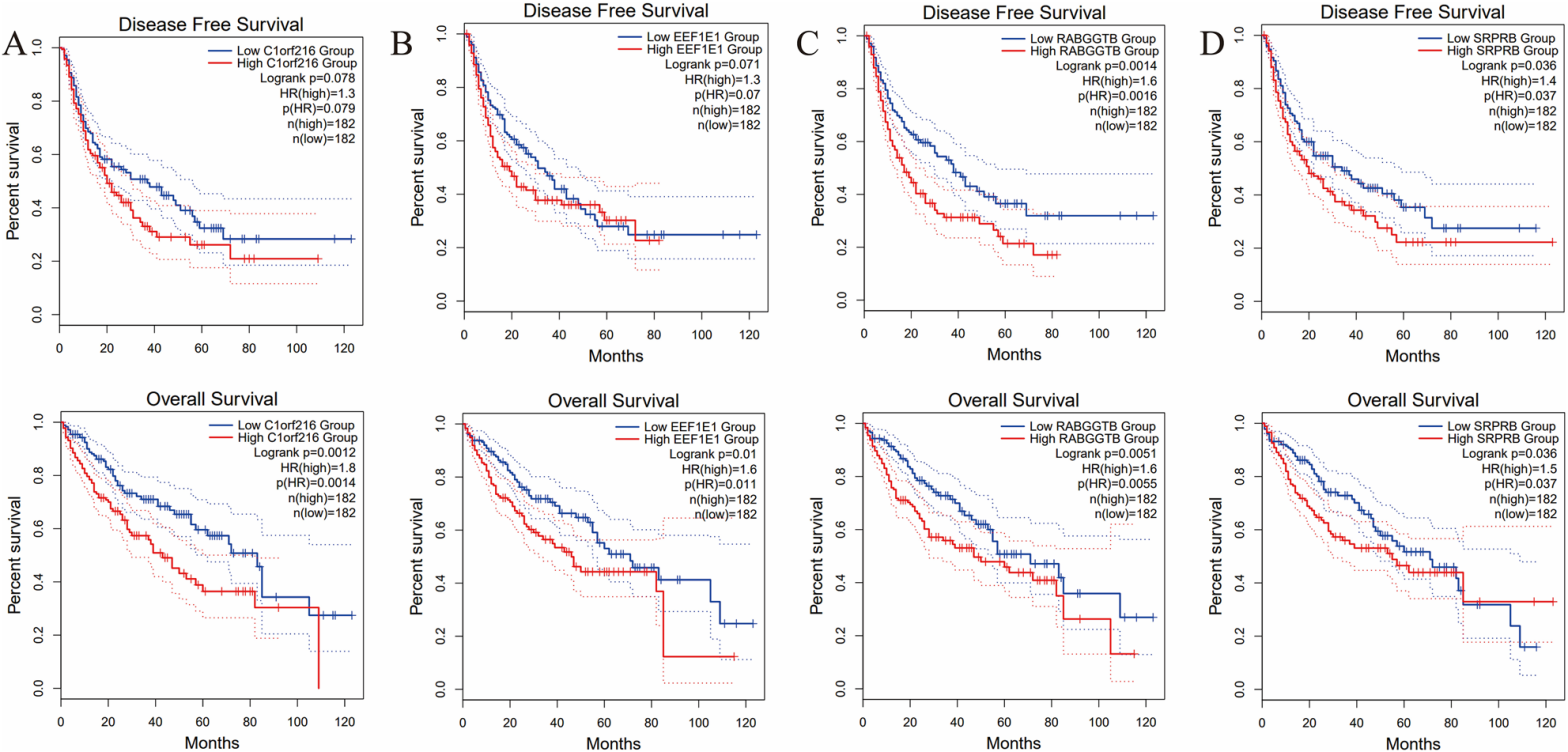
The correlation between the respective expression levels of the four SNRGs and the DFS and OS. (**A**) The Kaplan–Meier plot between the expression level of C1orf216 and DFS and OS in patients with HCC (*p* = 0.078 and *p* = 0.0012, respectively). (**B**) The Kaplan–Meier plot between the expression level of EEF1E1 and DFS and OS in patients with HCC (*p* = 0.071 and *p* = 0.01, respectively). (**C**) The Kaplan–Meier plot between the expression level of RABGGTB and DFS and OS in patients with HCC (*p* = 0.0014 and *p* = 0.0055, respectively). (**D**) The Kaplan–Meier plot between the expression level of SRPRB and DFS and OS in patients with HCC (*p* = 0.036 and *p* = 0.0036, respectively).

**Figure 4:**
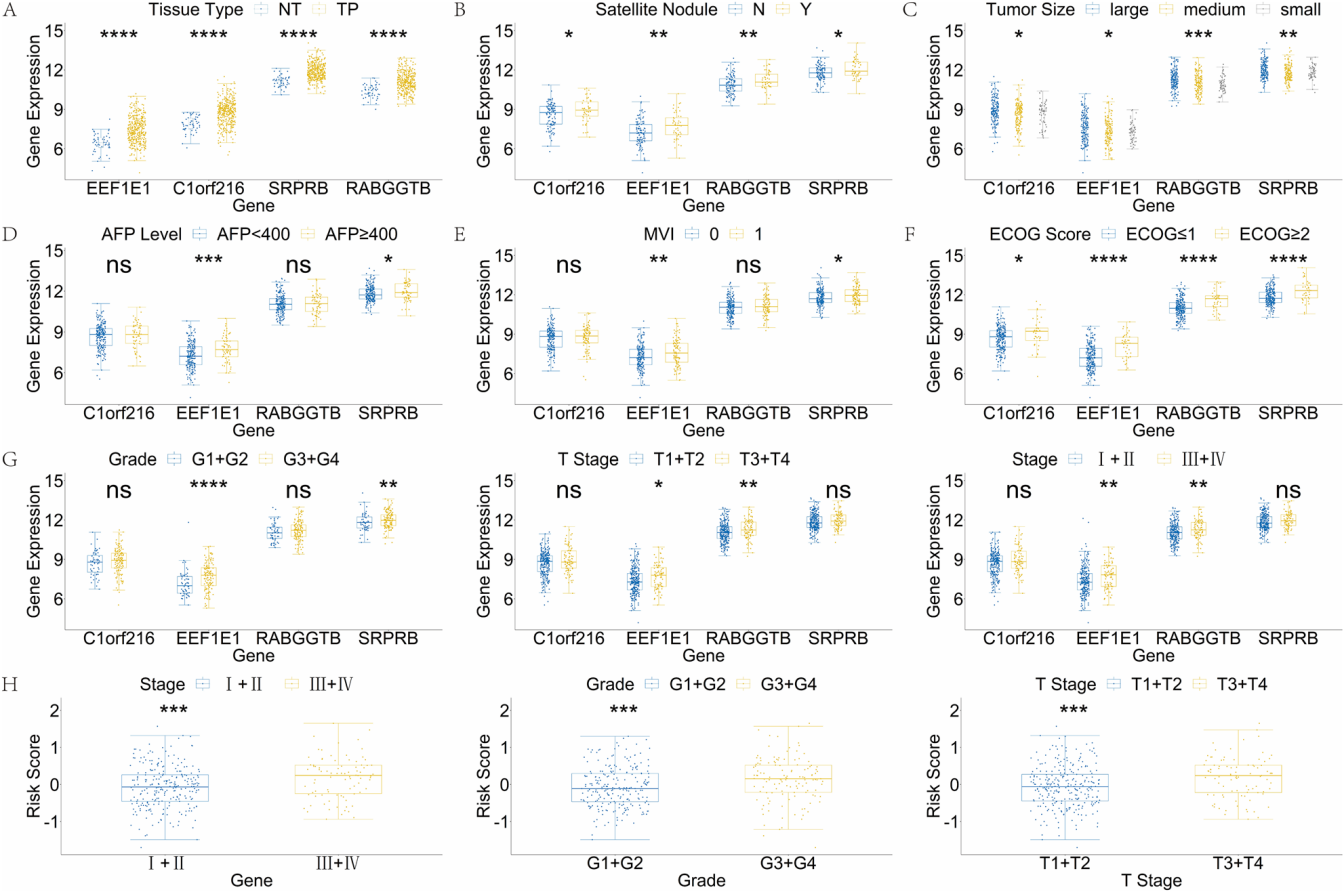
The correlation for pathobiochemical hallmarks of HCC and 4 SNRGs. (**A**) The correlation between gene expression and tissue type (NT: normal tissue, TP: primary tumor). (**B**)The correlation between gene expression and tumor with or without satellite nodules (N: without, Y: with). (**C**) The correlation between gene expression and tumor size. (D) The correlation between gene expression and AFP level. (**E**) The correlation between gene expression and MVI status (0: without MVI, 1: with MVI). (**F**) The correlation between gene expression and ECOG status (≤ 1 vs. ≥ 2). (**G**) The correlation between gene expression and tumor grade (G1 + G2 vs. G3 + G4), T stage (T1 + T2 vs. T3 + T4) and clinical stage (I + II vs. III + IV). (**H**) The correlation between risk score and tumor grade (G1 + G2 vs. G3 + G4), T stage (T1 + T2 vs. T3 + T4) and clinical stage (I + II vs. III + IV). NS: not significant; *p < 0.05, **p < 0.01, ***p < 0.001, ****p < − 0.0001.

To further validate the effectiveness of the model and the function of the four SNRGs, we conducted an analysis to examine the correlation between gene expression and various clinical characteristics. Initially, we examined the correlation between gene expression levels both DFS and OS among the four SNRGs individually. The Kaplan–Meier curves revealed a notable pattern: elevated expression of all genes integrated into the model was significantly linked to unfavorable OS outcomes (*p* < 0.05) among patients diagnosed with HCC (Fig. 3A–D). Elevated expression levels of RABGGTB and SRPRB were notably associated with a significant decrease in DFS (*p* < 0.05) (Fig. 3C,D). Additionally, increased expression of C1orf216 and EEF1E1 displayed trends of poor DFS, although the differences were not statistically significant (*p* = 0.078 and *p* = 0.071, respectively). Subsequently, the correlation for pathobiochemical hallmarks of HCC and the four SNRGs was further analyzed. For each gene, the expression levels in tumor tissue were significantly elevated compared to normal tissue (*p* < 0.0001) (Fig. 4A). Similarly, in tumor with satellite nodules, the expression levels were also significantly higher than those without satellite nodules (*p* < 0.05) (Fig. 4B). HCC tissues were categorized into three groups based on tumor size: large, medium, and small. The classification thresholds were set as follows: tumors with a maximum diameter of less than 3 cm were considered small, those with a diameter of 3 cm or more but less than 7 cm were considered medium, and tumors with a diameter of 7 cm or more were categorized as large. The outcomes of the Kruskal–Wallis test unveiled a significant correlation between high expression levels of C1orf216 and EEF1E1 (*p* < 0.05), high expression of SRPRB (*p* < 0.01), high expression of RABGGTB (p < 0.001), and larger tumor size within the HCC cohort (Fig. 4C). For patients with AFP level greater than 400U/ml or with MVI, a significant increase in the expression of EEF1E1 and SRPRB was observed (Fig. 4D–E). Besides, patients with worse physical conditions for which ECOG larger than 1 also displayed elevated expression levels of all four SNRGs (Fig. 4F). As for the most prevalent pathological characteristics: tumor grade, tumor T stage and tumor clinical stage, the high expression of EEF1E1 had significant relationship with worse grade and advanced stage (*p* < 0.0001 and *p* < 0.05, respectively) (Fig. 4G). Similar observations were made for RABGGTB and SRPRB (Fig. 4G). Furthermore, the correlation between risk score calculated by the four SNRGs and common pathological features was analyzed. As anticipated, HCC cases with high-risk scores were notably correlated with poor tumor grade and advanced T stage and clinical stage (*p* < 0.001) (Fig. 4H). The consistent alignment between the four SNRGs, the risk score, and the clinical-pathobiochemical characteristics of HCC further bolstered the reliability of the model.

### Validity of model in public datasets and optimization of model

**Figure 5:**
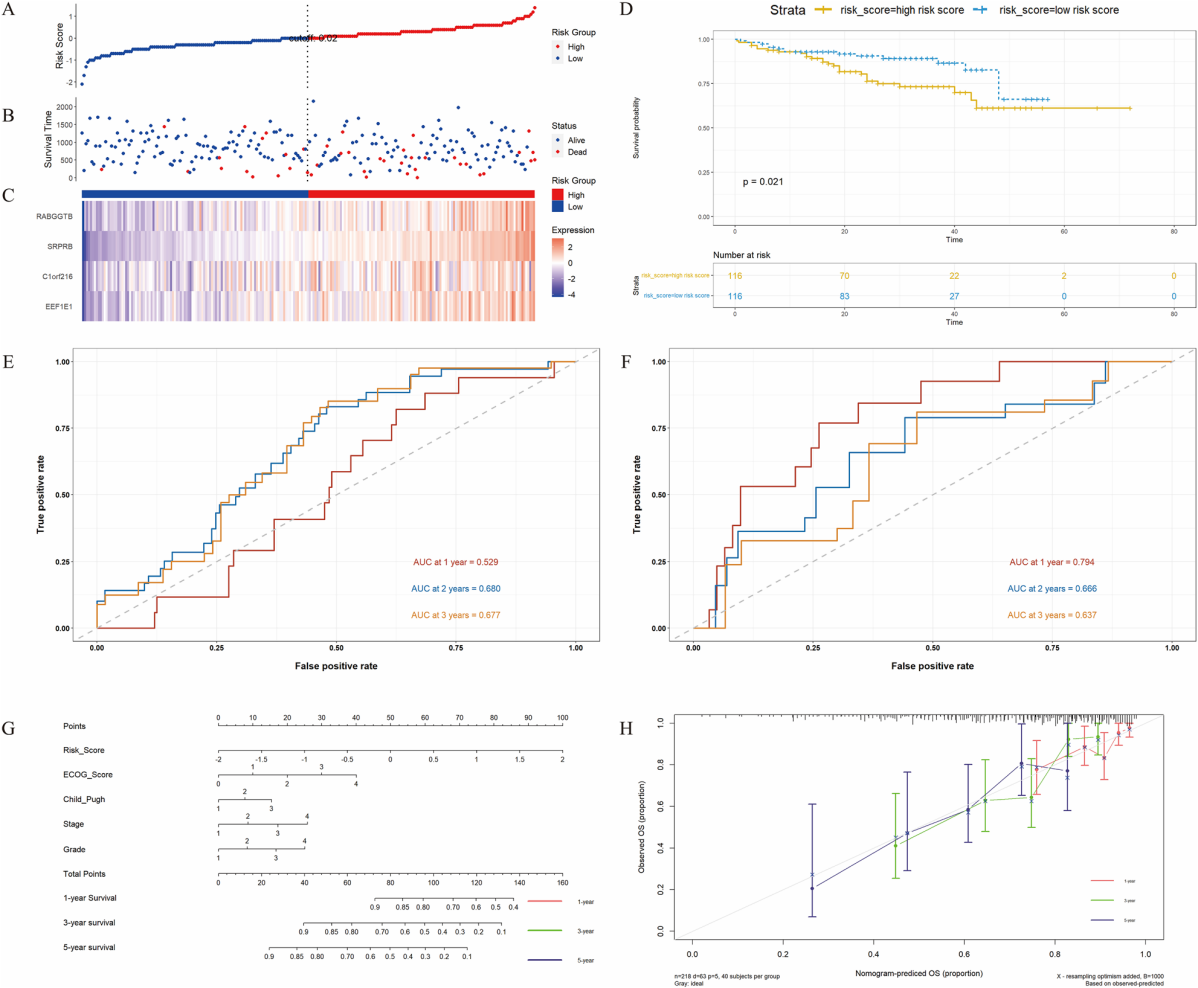
Validity of model in public datasets and optimization of model. (**A**) The risk score of each patient with HCC. (**B**) The patient survival based on the risk score. (**C**) The heat map of the three SNRGs in the high-risk group and the low-risk group. (**D**) The Kaplan–Meier plot of patients in a low- or high-risk group (*P* = 0.021), and the number of patients in different risk groups. (**E**) ROC curve analysis for the prognostic value of the prognostic model for different years. Data from ICGC (median risk score as the cut-off value). (**F**) ROC curve analysis for the prognostic value of the prognostic model for different years. Data from GSE76427 (median risk score as the cut-off value). (**G**) Nomogram model, with c-index = 0.708 (95% CI 0.645, 0.770). (**H**) The calibration curve of the nomogram model. Data from TCGA.

To further verify the universality of this model, the efficacy assessment of risk score for HCC was applied in public datasets: ICGC and GSE76427. The results showed the risk score of each patient with HCC in the ICGC database (Fig. 5A), patient survival based on the risk score (Fig. 5B), and the heat map of the three SNRGs in the high-risk group and the low-risk group (Fig. 5C). The survival time analyzed by Kaplan–Meier analysis in ICGC cohort could also be significantly distinguished by risk score (*p* = 0.021) (Fig. 5D). In both ICGC and GSE76427 cohorts, the ROC curve showed that the predictive model exhibited robust predictive capabilities, with AUC values were 0.529 (1 year), 0.680 (2 years) and 0.677 (3 years) (Fig. 5E); 0.794 (1 years), 0.666 (2 years), and 0.637 (3 years) (Fig. 5F) respectively. The consistency of all the aforementioned data with the results obtained from the TCGA cohort serves to further validate the universal applicability and reliability of the predictive model.

To further refiner the model, clinical pathobiochemical hallmarks were incorporated into the model. We introduced major clinical pathobiochemical hallmarks ECOG score, Child–Pugh score, tumor stage and tumor grade into the model and constructed a nomogram model, with a c-index 0.708 (95% CI 0.645, 0.770) (Fig. 5G). Moreover, the calibration curve confirmed the reliability of this optimized model, portraying a strong predictive capacity (Fig. 5H).

### The correlation between immune infiltration and SNRGs

**Figure 6:**
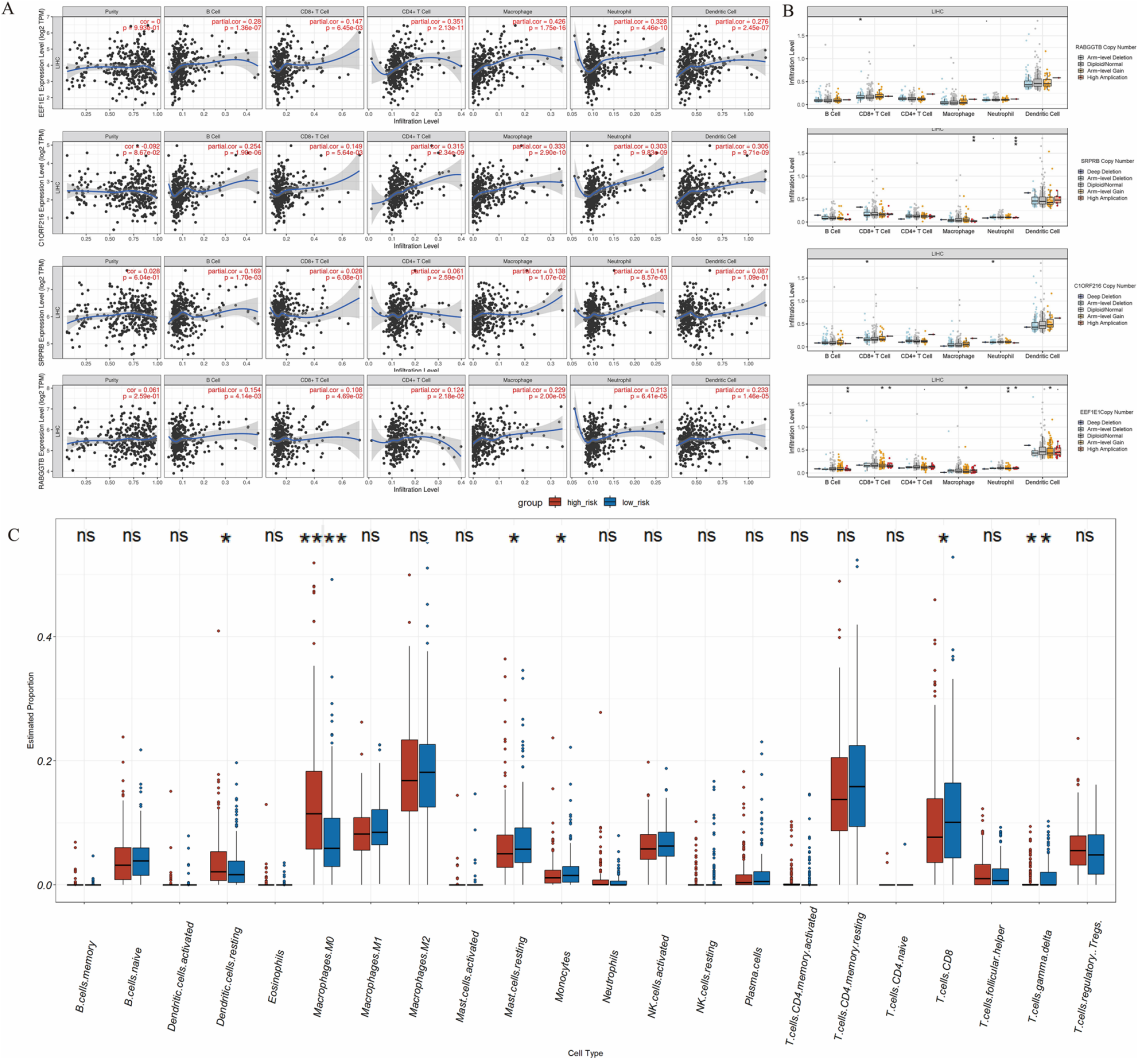
The correlation between immune infiltration and SNRGs: (**A**) the correlation between the expression of four SNRGs and immune infiltration. (**B**) the correlation between copy number of four SNRGs and immune infiltration. (**C**) the correlation between risk score and immune infiltration.

Given that immunotherapy holds substantial significance in HCC treatment, we proceeded to delve into the correlation between immune infiltration and SNRGs. The expression of SNRGs had significant correlation with immunosuppressive cells such as macrophage and neutrophil (Fig. 6A). Among the four SNRGs, EEF1E1 exhibited the most pronounced correlation with macrophage and neutrophil infiltration. The partial correlation coefficients stood at 0.426 (*p* < 0.0001) and 0.328 (*p* < 0.0001), respectively (Fig. 6A). The variation of copy number for the four SNRGs also showed significant correlation with immune cell infiltration (Fig. 6B). To further distinguish the subpopulations of infiltrated immune cells in high- and low- risk group in HCC, R package “immunedeconv” was used to analyze immune cell subpopulation. As expected, macrophages M0 and dendritic cells. resting which play an immunosuppressive role were significant high in high-risk group. Conversely, immune cell populations with anti-tumor properties, such as CD8 + T cells and γδT cells, were significantly diminished in the high-risk group (Fig. 6C).

## Discussion

The advancement of high-throughput sequencing technology has led to the proliferation of tumor prediction models. In HCC, multidimensional prediction models have been devised to forecast patients' prognoses by incorporating various omics data, including genomics, transcriptomics, proteomics, metabolomics, epigenomics, and gut microbiota^18–22^. The development of multidimensional prediction models holds significant importance in forecasting patient prognosis. While tumorigenesis and progression result from genetic and epigenetic disruptions^23,24^, the clinical manifestation of tumor progression primarily presents by adverse clinical pathological features, ultimately facilitating tumor progression or metastasis. Thus, it is crucial to investigate the mechanisms leading to the emergence of adverse clinical pathological features and predict their occurrence. Presently, research in HCC to predict clinical feature predominantly concentrates on MVI. Imaging omics and transcriptomics can effectively predict whether HCC patients will develop MVI^25,26^, but research on satellite lesions is limited.

Satellite nodule is a significant biomarker of early recurrence and poor prognosis of HCC^11,12^. In recent study, the presence of satellite nodules [HR 3.07; 95% CI 1.14–8.24] was identified as independent factors associated with tumor recurrence^27^. Besides, some genes which have close relationship with tumor metastasis such as choroideremia-like (CHML) and Insulin-like growth factor II mRNA-binding protein 3 (IMP3) are more abundant in satellite nodules^28,29^. All these evidences indicate that satellite nodule is a key biomarker of HCC, and HCC with satellite nodules may exhibit a distinct gene expression profile when compared to HCC without satellite nodules. Consequently, constructing a prognostic model based on these distinct genes could potentially yield an enhanced predictive value for individuals diagnosed with HCC.

As satellite nodules are a significant pathological factor in intrahepatic metastasis of HCC, understanding the mechanisms involving these genes is of direct interest. Intrahepatic metastasis of HCC is a complex process closely associated with aberrant activation of cellular pathways and the tumor microenvironment. Commonly observed activations of the Wnt/β-catenin pathway, Hippo pathway, and EMT processes can lead to intrahepatic tumor spread^30–32^. Furthermore, alterations in the extracellular matrix within the tumor microenvironment, including changes in matrix stiffness, cell adhesion, vasculogenic mimicry, drug delivery, and the immunosuppressive microenvironment, strongly facilitate HCC tumorigenesis and metastasis^33,34^. In this study, we utilized the TCGA original scanned pathological data, accessible through the official website of the Genomic Data Commons, to identify HCC patients with satellite nodules and to record tumor sizes. Following the categorization of patients into three groups—those with satellite nodules, those without satellite nodules, and those in an undefined category—the differentially expressed genes within the first two groups were subjected to analysis. In HCC with satellite nodules compared with without satellite nodules, 253 DEGs up-regulated and 86 DEGs down-regulated were identified. To discern the malignant characteristics of satellite nodules, a set of 253 up-regulated DEGs were subjected to GO and KEGG analyses. The GO and KEGG analyses revealed that the SNRGs were primarily associated with processes such as focal adhesion, cell-substrate junction cell adhesion, and pathways related to the extracellular matrix. These findings collectively suggest that HCC with satellite nodules exhibits a heightened potential for invasion and metastasis. Additionally, the KEGG analysis highlighted the enrichment of metabolism-related pathways and pathways associated with tumor immunity within the SNRGs. These findings suggest that the DEGs we have identified may contribute to the occurrence of satellite nodules and intrahepatic metastasis by promoting extracellular matrix alterations and enhancing tumor cell activity.

Through univariate Cox regression analysis, numerous genes were identified that exhibited significant associations with the OS of HCC patients. Among these, 8 SNRGs were selected for further investigation via LASSO regression analysis, after being matched with the DEGs obtained from the comparison between HCC tumor tissues with or without satellite nodules. After analysis, four genes (RABGGTB, SRPRB, C1orf216 and EEF1E1) were used to construct the predictive model. Within this set of four genes, SRPRB and EEF1E1 have been unveiled as pivotal players in the context of HCC. SRPRB also known as APMCF1, SR-beta, is a novel human gene whose transcript is upregulated in apoptotic MCF-7 cells^35^. Its expression was found to be up-regulated in tumor tissues when compared to corresponding normal tissues in cases of liver, colon, esophagus, lung, and breast carcinomas^36^. SRPRB, alongside T-cell differentiation protein (MAL), diphosphoinositol polyphosphate phosphohydrolase type 2 (NUDT4), plakophilin 4 (PKP4), and signal sequence receptor (SSR1), constituted the five most prominent genes implicated. These genes exhibited significant up-regulation in instances of short-term survivors when contrasted with long-term survivors and early-stage cases within ovarian cancers.^37^. Within the realm of HCC, SRPRB has been integrated into several prognostic models, including those grounded in endoplasmic reticulum stress-related genes and the activity fluctuations of hallmark genes.^38,39^. Nevertheless, the precise biological role of SRPRB in the context of HCC necessitates further exploration and investigation. EEF1E1, also recognized as P18 and AIMP3, represents one of the aminoacyl-tRNA synthetases, a vital class of enzymes endowed with an evolutionarily conserved mechanism pivotal for protein synthesis.^40^. In HCC, EEF1E1 mRNA and protein expression in tumor was statistically higher than normal tissue. The EEF1E1 protein level was positively correlated to the CD3, CD4, PD1 and was negatively correlated to the CD8. The expression level of EEF1E1 in HCC was significantly correlated with the key genes involved in the p53 pathway^41^. However, AIMP3 haploinsufficiency disrupted oncogene-induced p53 activation and genomic stability. In AIMP3 heterozygous cells, cells became susceptible to cell transformation induced by oncogenes such as Ras or Myc alone. From this perspective, AIMP3 plays crucial roles in p53-mediated tumor-suppressive response against oncogenic stresses^42^.

An effective model should exhibit a strong correlation with real-world clinical applications. After the predictive model constructed, the relationship between four SNRGs and clinical parameters was analyzed. These four SNRGs exhibited notable correlations with key clinical parameters, including tumor size, AFP level, microvascular invasion (MVI), ECOG score, and tumor grade. By computing a risk score for each HCC patient using the expression coefficients of these four genes, and subsequently utilizing the median risk score as a threshold to categorize patients into either a high-risk group or a low-risk group, a significant differentiation in OS between the two groups can be achieved. The ROC curve also showed this model had effective separability. External datasets further substantiated the reliability and credibility of the model. Clinical variables including ECOG score, Child–Pugh score, tumor stage, and tumor grade were integrated into the model to amplify its practical clinical utility. These variables are presented in the form of a nomogram, enhancing its applicability. The c-index (with a value of 0.708) along with the calibration curve serve to reinforce the model's consistency and reliability. During our continued investigation, we discovered that patients with elevated risk scores also exhibited heightened levels of infiltration by immunosuppressive cells and reduced levels of infiltration by immune-killing cells. This observation suggests a potentially close association between this phenomenon and the metastasis and recurrence of HCC with satellite nodules.

In summary, we constructed and validated a prognostic model for patients with HCC based on satellite nodules-related genes. The SNRGs comprising the model demonstrated a strong correlation with clinical parameters. Furthermore, the risk score computed by the model effectively discriminated between patients with favorable and unfavorable prognoses. Undoubtedly, our research did have certain limitations. Owing to the absence of clinical samples, our study had to rely solely on databases for its execution. Even though multiple databases were employed in this study, including TCGA, ICGC, and GEO, the absence of experimental validation still imposed constraints on the precision of the outcomes. Additionally, since the pathological information was sourced from TCGA raw materials, certain pathological descriptions were found to be inaccurate. This discrepancy resulted in a reduction in available data and subsequently rendered sequencing data unavailable for numerous patients. Furthermore, the potential molecular mechanisms underlying the four genes utilized for modeling lacked comprehensive validation through in vivo or in vitro functional experiments.

### Supplementary Information

Supplementary Information 1.

Supplementary Information 2.

Supplementary Information 3.

Supplementary Information 4.

## Data Availability

Publicly available datasets were analyzed in the study. The RNA-seq data of TCGA_LIHC, ICGC_ LIRI_JP and GSE76427 were separately from https://portal.gdc.cancer.gov/, https://daco.icgc.org/ and https://www.ncbi.nlm.nih.gov/geo/query/acc.cgi?acc=GSE76427 .
