# Supplementary figures and images for: Identification and validation of a prognostic model based on four genes related to satellite nodules in hepatocellular carcinoma
Source: Sci Rep. 2024 Jul 7;14:15633. doi: 10.1038/s41598-024-66610-z (PMC11228042; doi:10.1038/s41598-024-66610-z)

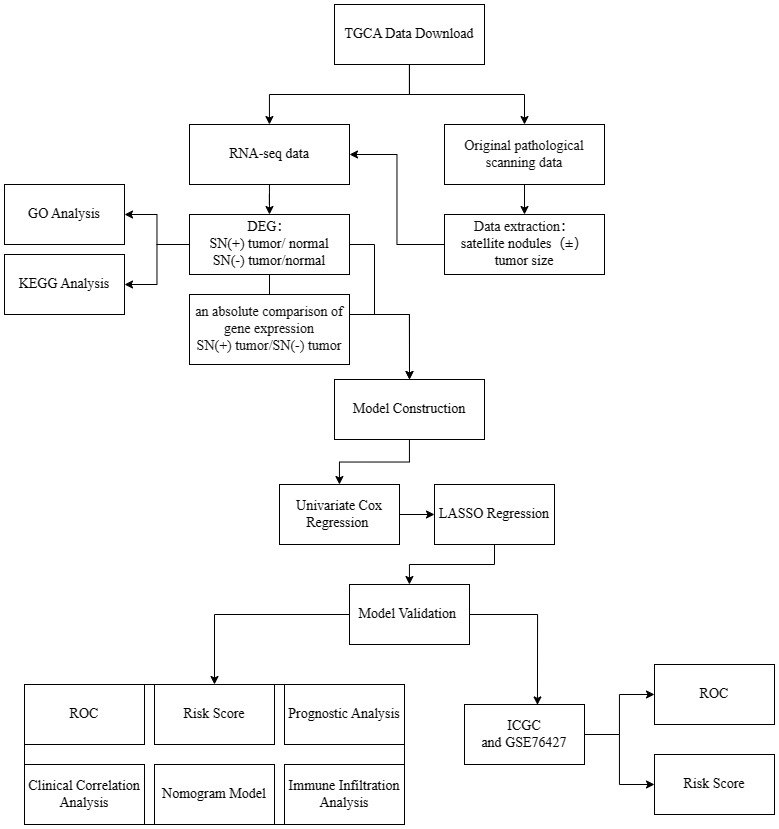

Supplement: Supplementary file 1 — Supplementary Information 1. [file 41598_2024_66610_MOESM1_ESM.jpg]
